# Growth performance, enteric methane emissions, and economic impact of alternative feeding strategies for Simmental fattening bulls

**DOI:** 10.1093/tas/txaf043

**Published:** 2025-04-05

**Authors:** Christian Koch, Manfred Schönleben, Jason J Hayer, Joachim Mentschel, Norbert Göres, Paolo Fissore, Katrin Gnjidic, Max Görtz, Hermann Bischoff, Josef Bauerdick, Helga Sauerwein, Morteza H Ghaffari

**Affiliations:** Educational and Research Centre for Animal Husbandry, Hofgut Neumühle, Muenchweiler a.d. Alsenz 67728, Germany; Sano - Moderne Tierernährung GmbH, Grafenwald 1, Loiching 84180, Germany; Sano - Moderne Tierernährung GmbH, Grafenwald 1, Loiching 84180, Germany; Sano - Moderne Tierernährung GmbH, Grafenwald 1, Loiching 84180, Germany; Sano - Moderne Tierernährung GmbH, Grafenwald 1, Loiching 84180, Germany; Sano - Moderne Tierernährung GmbH, Grafenwald 1, Loiching 84180, Germany; Sano - Moderne Tierernährung GmbH, Grafenwald 1, Loiching 84180, Germany; Sano - Moderne Tierernährung GmbH, Grafenwald 1, Loiching 84180, Germany; Sano - Moderne Tierernährung GmbH, Grafenwald 1, Loiching 84180, Germany; Sano - Moderne Tierernährung GmbH, Grafenwald 1, Loiching 84180, Germany; Faculty of Agricultural, Nutritional and Engineering Sciences, Institute of Animal Science, Physiology Unit, University of Bonn, Bonn 53111, Germany; Faculty of Agricultural, Nutritional and Engineering Sciences, Institute of Animal Science, Physiology Unit, University of Bonn, Bonn 53111, Germany

**Keywords:** beef cattle, by-product feeding, food-feed competition, laser-methane-detector, methane mitigation, sustainable livestock production

## Abstract

This study evaluated the effects of conventional silage-based (CONVL) and byproduct-based (ByProd) TMR, along with a tannin-seaweed supplement (ClimaSAN; a 50:50 proprietary blend of hydrolyzable tannins from chestnut (*Castanea sativa*) and brown seaweed (*Ascophyllum nodosum*), Sano GmbH, Loiching, Germany), on performance, enteric methane emissions, and economic outcomes. A 306-d trial was conducted with 32 Simmental bulls assigned to 2 treatments (*n = *16/group) formulated using the NASEM (National Academies of Sciences, Engineering, and Medicine). 2016. Nutrient requirements of beef cattle. 8th revised ed. The National Academies Press, Washington, DC, USA. https://doi.org/10.17226/19014) guidelines, in conjunction with the CNCPS (v6.5.5). The primary differences between ByProd and CONVL diets were neutral detergent fiber (aNDFom: 31.6% vs. 33.3% of dry matter [DM]), physically effective NDF (peNDF: 16.6% vs. 21.9% DM), acid detergent fiber (ADF: 16.5% vs. 19.1% DM), sugar content (8.24% vs. 4.26%), and ether extract (EE: 2.85 vs. 4.03% DM). From days 155 to 241, both groups were supplemented with ClimaSAN at 6 g/kg of TMR DM. Enteric methane emissions were measured using a portable laser methane detector (Mini-Green® Tokyo Gas Engineering Solutions, Ltd., Tokyo, Japan). Data were analyzed using a repeated-measures model in SAS 9.4, with treatment, time, and their interaction as fixed effects, and pen and animal as random effects. Bulls fed the ByProd TMR consumed less metabolizable energy, fibrous carbohydrates (including aNDFom, peNDF, and ADF) and ether extract (*P* < 0.05) but significantly more sugar and non-fiber carbohydrates (*P *< 0.01). However, the treatments did not significantly affect average daily gain (ADG), the ADG/metabolizable energy intake ratio and methane emissions. Methane data were categorized into three phases: pre-supplementation (Days 29 to 135), during ClimaSAN (Days 155 to 241), and post-supplementation (Days 263 to 306). ClimaSAN reduced methane by 9.82% in Period 2, and despite a 1.42% increase in Period 3, emissions remained below baseline. Carcass revenue (€4.61 vs. €4.60/kg, *P* = 0.80) and dressing percentage (57.0% vs. 56.8%, *P* = 0.71) were unaffected by dietary treatment. Economic performance was favorable for both diets, with the ByProd TMR group achieving a higher income over feed cost (€1,221 vs. €1,187/head, *P* = 0.11) and a higher profit margin (€371 vs. €337/head, *P* = 0.11). ClimaSAN-supplemented diets reduced methane by 9.82% with lasting post-supplementation effects. In summary, rations based on byproducts and co-products can reduce feed costs in cattle farming without affecting production efficiency.

## Teaser

This study demonstrates that byproduct-based diets in Simmental bulls can significantly reduce feed costs without compromising growth performance while also contributing to methane emission reductions. Supplementing with a tannin-seaweed blend reduced methane emissions by nearly 10%, offering a sustainable approach to both economic efficiency and environmental impact in cattle farming.

## Graphical Abstract

**Graphical Abstract:**
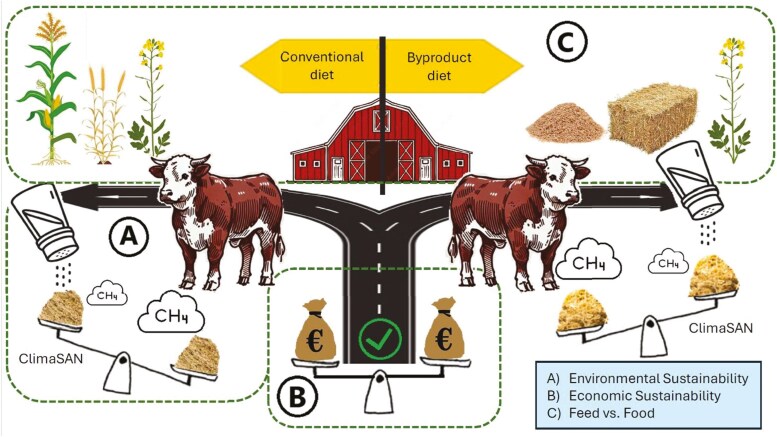

## INTRODUCTION

In Europe, approximately 80% of beef production is derived from dairy, beef-on-dairy, or dual-purpose breeds, with Simmental bulls accounting for nearly 50% of Germany’s total beef output (Schütte et al., 2021). Among the top beef-producing countries in Europe, France, Germany, and the United Kingdom lead the industry (Hocquette et al., 2018). Notably, the carbon intensity of beef from specialized beef herds is almost four times higher than that from dairy herds (Gerber et al., 2013). In Western Europe, particularly Germany, bulls are typically fattened in intensive systems that rely on maize silage along with energy-rich grains, like wheat or protein-rich byproducts, like brewers’ grains sourced locally or from imported oilseeds, like full-fat soybean (Schütte et al., 2021). However, increasing competition through biogas digesters, using maize silage as substrate, coupled with the expected decline in crop yields due to climate change (Jägermeyr et al., 2021), is reducing the availability of silage and threatening the profitability of cattle fattening operations (Henke and Theuvsen, 2013). Consequently, there is growing interest in alternative feedstuffs such as straw and byproducts (Koch et al., 2023).

By-product-based concentrates offer significant environmental advantages, including reduced land use, lower carbon footprints, and decreased eutrophication potential (Lindberg et al., 2021). Additionally, they help address the growing challenge of feed vs. food competition by utilizing co-products from human food production, thereby reducing pressure on resources that could otherwise be used for direct human consumption. As alternative feedstuffs become more significant and available, it is essential to design feeding rations that meet the animal’s nutritional needs, support rumen function, and contribute to the sustainable reduction of enteric methane (CH₄) emissions. Reducing CH₄ emissions is critical for mitigating climate change since CH₄ is the second most impactful greenhouse gas after carbon dioxide, with a much higher heat-trapping capacity (Hristov et al., 2022). Around 30% of global CH₄ emissions are attributed to enteric fermentation in ruminant livestock, and within agriculture, about 81% of CH₄ emissions come from livestock, with beef cattle contributing 35% and dairy cattle 30% (Opio et al., 2013; FAO, 2023). Enteric CH₄ accounts for 41% of livestock-related greenhouse gas emissions, and the livestock sector as a whole is responsible for 14.5% of global anthropogenic emissions (Gerber et al., 2013; FAO 2023). While increasing dietary concentrates can reduce CH₄ emissions, this approach again raises concerns about feed competition, availability, and rumen health (Hristov et al., 2022). Strategies to mitigate CH₄ emissions in dairy and beef cattle, as well as small ruminants, include the use of CH₄ inhibitors, alternative electron sinks, vegetable oils, oilseeds, algae, and tannin-containing feeds, but still lack widespread application, with CH₄ emission estimates still mainly relying on book values (Hristov et al., 2022; McGurrin et al., 2023). So far, the high cost and complexity of data collection has limited the inclusion of CH₄ measurements in daily animal management. Respiration chambers are the gold standard for CH₄ measurement. Nonetheless, they are expensive, labor-intensive, and disrupt animal behavior, making them unsuitable for large-scale studies (Sorg et al., 2018). More affordable, noninvasive alternatives have emerged, such as the GreenFeed emission monitoring system and laser CH₄ detectors (LMD). These systems capture gas concentrations from the animal’s breath to measure CH₄ emissions (Ryan et al., 2022). Research by Sorg et al. (2018) and Sorg (2022) has demonstrated that LMDs provide reliable, precise CH₄ data in real-world farm settings without interfering with animal behavior, making them efficient for large-scale monitoring.

As discussions on agricultural CH₄ mitigation evolve, greater emphasis should be placed on feed availability and developing innovative feeding strategies for bull fattening. The goal of this study was to evaluate the growth performance, enteric CH₄ emissions, and economic outcomes of feeding Simmental bulls either a conventional silage-based (CONVL) or byproduct-based (ByProd) total mixed ration (TMR) until they reached a final body weight of around 750 kg. Additionally, the study explored the impact of a tannin- and seaweed-based anti-methanogenic supplement administered over a three-month period in both diets. The ByProd TMR was formulated to contain similar levels of rumen-fermentable carbohydrates as the CONVL diet but with lower concentrations of structural components such as neutral detergent fiber (aNDFom), acid detergent fiber (ADF), crude fiber (CF), and physically effective neutral detergent fiber (peNDF). We hypothesized that both feeding strategies would result in comparable growth rates and economic outcomes but differ in their potential to mitigate CH₄ emissions.

## MATERIAL AND METHODS

### Animals and Management

The animal feeding experiment took place between April 2022 and February 2023 at the Educational and Research Centre for Animal Husbandry, Hofgut Neumühle, Germany. All experimental procedures received approval from the Animal Ethics Committee of the Department for Animal Welfare Affairs (Landesuntersuchungsamt Rheinland-Pfalz, Koblenz, Germany) in accordance with the German Animal Welfare Act. Thirty-two Simmental bulls were stratified by body weight (BW) and randomly assigned to one of two feeding groups: CONVL and ByProd (*n* = 16 per group; 4 pens per group, 4 bulls per pen). Initial BW (Ø = 209 kg ± 9 kg) was consistent across both groups. Each pen (3.93 m² per bull) was outfitted with slated floors, rubber mats, and two pressure bowls for free access to water. Diets were formulated following NASEM (2016) guidelines, designed to provide sufficient energy and protein for a 500 kg fattening bull, with a target daily weight gain of approximately 1.70 kg per animal. Bulls were weighed monthly to monitor growth.

### Dietary Treatments and Diet Formulation

The ByProd diet primarily consisted of co- and byproducts from human food production, including beet pulp, wheat straw, brewers’ grains, wheat bran, canola meal, and a vitamin-mineral premix. A key difference between the two diets was the ~70% lower inclusion of forage silage (maize and grass) in the ByProd diet compared to CONVL. Dry matter intake (DMI) was calculated as the total feed consumed by each pen of four bulls, expressed in kilograms of dry matter per day. DMI was measured daily, providing precise values per pen, which were used as covariates in individual animal CH₄ measurements, as well as in the calculation of average daily gain (ADG) and feed conversion rate (FCR). The dry matter (DM) content of the CONVL TMR averaged around 45.0%, compared to 52.1% in the ByProd TMR (Table 1). Both diets exhibited similar nutritional parameters per kg DM, such as metabolizable energy (ME), starch, crude protein (CP), non-fiber carbohydrates (NFC), and acid detergent lignin (ADL). The ByProd TMR was formulated to support a growth rate comparable to the CONVL diet (NASEM, 2016). To prevent feed sorting, wheat straw was pre-chopped to a theoretical length of 20 mm, and a 10% sugar beet molasses/water mixture was added to the ByProd TMR. Both groups received the tannin-seaweed complex (ClimaSAN; Sano GmbH, Loiching, Germany) at 6 g/kg of TMR dry matter from days 155 to 241. Bulls were slaughtered after 306 days of fattening. Daily nutrient intake, including DM, ME, and CP, was recorded for each pen of four bulls. Every morning at 6:30 a.m., the pen-specific remains were removed and recorded. Then, between 7:30 and 8:30 am, the pen-specific feed was weighed and administered ad libitum. The average daily DM feed intake per bull was then calculated by subtracting the previous day’s fed amount from the recorded remains for the specific pen, dividing the net amount by the number of bulls per pen (n = 4) and multiplying by the DM content of the feed treatments. Laboratory analyses assessed nutrient and peNDF intake, complemented by evaluations of feed particle length.

**Table 1.** Ingredients and chemical composition of the experimental diets (ByProd and CONVL), including commodity market prices following 2022 board of trade information and average silage production costs

Feeds (Amount in kg as fed)	Treatments		Investment
	ByProd	CONVL	
Corn silage (season 2021)^a^	5.00	10.00	50 €/t
Gras silage (season 2022)^a^	-	6.00	50 €/t
Beet pulp silage (season 2022)^a^	2.00	-	40 €/t
Brewers’ grains (season 2022)^a^	3.00	-	30 €/t
Wheat straw^b^	1.00	0.50	80 €/t
Wheat bran^b^	0.60	-	150 €/t
Wheat gluten feed^b^	0.60	-	250 €/t
Maize grain ground^b^	2.00	1.80	250 €/t
Barley grain ground^b^	2.00	2.40	230 €/t
Solvent-extracted canola meal^b^	0.60	1.40	350 €/t
Beet molasses mix (liquid)^b^	2.00	-	110 €/t
Bumisan CLIMA^3c^	0.20	0.20	800 €/t
Acid Protect TMR^1c^	0.05	0.05	1,700 €/t
∑ kg As Fed (DM)	19.05 (9.93)	22.35 (10.07)	
Investment per animal*day	2.38 €	2.58 €	
Dietary composition (Dry matter basis)^2^			
Dry matter (%)	52.1	45.1	
ME, MJ/kg	11.1	11.4	
Crude fiber (%)	10.1	15.3	
aNDFom (%)	31.6	33.3	
ADF (%)	16.5	19.1	
ADL (%)	2.80	2.91	
peNDF (%)	16.6	21.9	
NFC (%)	45.5	43.1	
Sugar (WSC) (%)	8.24	4.26	
Starch (%)	30.6	30.4	
Soluble Fiber (%)	4.67	4.27	
uNDF (%)	7.63	7.57	
Crude protein (CP) (%)	12.9	13.7	
Soluble protein (%)	4.11	4.97	
Ether Extract (EE) (%)	2.85	4.03	
Ash (%)	6.27	5.94	
Ca (%)	0.64	0.61	
P (%)	0.46	0.42	
Mg (%)	0.28	0.24	
Na (%)	0.24	0.20	

^1^Feed preservative (Propionic-acid, Sorbic-acid, sodium (Na), and calcium (Ca) propionate).

^2^DM (dry matter), ME (metabolizable energy, MJ/kg), CF (crude fiber), aNDFom (neutral detergent fiber, organic matter basis, exclusive of ash), ADF (acid detergent fiber), ADL (acid detergent lignin), peNDF (physically effective neutral detergent fiber), NFC (non-fiber carbohydrates), WSC (water-soluble carbohydrates, sugar), starch (nonstructural carbohydrate component), soluble fiber (fiber fraction soluble in neutral detergent), uNDF (undigested neutral detergent fiber), CP (crude protein), soluble protein (fraction of crude protein soluble in buffer solutions), EE (ether extract, lipid content), P (Phosphorus), and Mg (Magnesium).

^3^Professional bull-fattening mineral and vitamin premix, composed per kg of 19.5% Ca, 0.5% P, 6.5% Na, 2.0% Mg, 4.000mg Zn, 2.500mg Mn, 600mg Cu, 60mg I, 18mg Se, 210.000 I.U. Vit. A, 60.000 I.U. Vit. D, 360mg Vi.t. E, 180mg Vit. B_1_.

^a^Economic evaluation following average silage gross production costs, free feed-bunk (KTBL 2009).

^b^Board of Trade Kassa market price information 2022 (Statista 2024; https://de.statista.com).

^c^Gross market sales prices 2022.

### ClimaSAN Supplementation

During the feeding trial, both dietary groups received ClimaSAN (Sano GmbH, Loiching, Germany), a proprietary 50:50 mixture of hydrolyzable tannins from chestnut (*Castanea sativa*) and brown seaweed (*Ascophyllum nodosum*) extracts. ClimaSAN is formulated to reduce enteric CH₄ emissions by modulating rumen fermentation. The mechanism is based on the bioactive properties of hydrolyzable tannins, which can form a complex with proteins and carbohydrates to inhibit the activity of methanogenic archaea, as well as the bioactive compounds in brown seaweed extracts, which are thought to suppress CH₄ production (Goel and Makkar, 2012; Cardoso‐Gutierrez et al., 2021; Beauchemin et al., 2022). The administered dose of 6 g ClimaSAN per kg TMR DM from day 155 to 241 was chosen based on preliminary trials and literature (Goel and Makkar, 2012; Machado et al., 2015; Cardoso‐Gutierrez et al., 2021; Koch et al., 2023) showing that a reduction in CH₄ emissions can be achieved without negative effects on feed intake or animal performance. While the ClimaSAN supplement was thoroughly mixed into the respective TMR to ensure even distribution, feed intake was measured continuously on a daily basis throughout the trial.

### Animal Health

Veterinarians at the research farm conducted weekly health assessments. These included evaluations of behavior, general appearance, and visible health indicators such as locomotion and respiratory traits. Animals displaying suspicious symptoms underwent a complete clinical examination. Additional veterinary checks were conducted during monthly weigh-ins. No clinical abnormalities were observed during the experimental period.

### Sampling and Laboratory Analyses

Samples of silage, CONVL, and ByProd TMR (800 g each) were collected, vacuum-sealed, and sent to the Sano CVAS laboratory in Popovaca, Croatia, for nutrient analysis. Samples were air-dried, using the forced convection approach at 60 °C for 24 h, and then ground. Nutrient degradation metrics were obtained via in vitro analyses following standard procedures of the Cornell Net Carbohydrate and Protein System (CNCPS), employing CVAS European NIR calibrations. In brief, dried and uniformly ground substrate samples were incubated in buffered rumen fluid under controlled anaerobic conditions. This formed the basis for the EU NIR calibration to accurately quantify regional feed-specific degradation parameters and nutrient concentrations.

### Particle Separation

Monthly duplicate samples of the two TMRs were collected from the feed bins of each group to assess particle size distribution. Samples were separated using a 3-sieve particle separator (Wasserbauer Shaky 4.0 model; WPS) with sieve diameters of 19 mm, 8 mm, and 4 mm. The WPS separated particles into four fractions: long (>19 mm), medium (8 to 19 mm), short (4 to 8 mm), and fine (<4 mm). These fractions were vacuum-sealed and sent to Sano Cumberland Valley Analytical Services (CVAS) in Popovaca, Croatia, for nutrient analysis using near-infrared reflectance spectroscopy (NIR) and proprietary European NIR calibrations. The physical effectiveness factor (pef) was calculated as the percentage of DM retained on the top three sieves (Yang and Beauchemin, 2006). The sorting index, which indicates how selectively the bulls consume (or avoid) certain particle fractions, was calculated according to Leonardi and Armentano (2003). The calculation of peNDF was performed by multiplying the NDF content of the TMR by the pef. The particle size distribution for the experimental feed is presented in Table 2.

**Table 2.** Particle size distribution of experimental diets

Sieve/Pan (%)	Treatments		SEM	*P*-value
	ByProd	CONVL		
> 19 mm	0.44	0.47	0.32	0.93
8 – 19 mm	21.8	31.9	2.39	<0.001
4 – 8 mm	25.8	21.3	2.15	0.05
< 4 mm	51.9	46.3	2.75	0.05

### Measurement of Enteric CH₄ Emissions

CH₄ concentration profiles (ppm × m) were measured using a laser CH₄ detector (LMD; Mini-Green® Tokyo Gas Engineering Solutions, Ltd., Tokyo, Japan). Two recording sessions were conducted daily to capture the diurnal pattern of enteric CH₄ emissions: one in the morning (around 10:00 am), which began shortly after feeding, and one in the afternoon, which occurred between 1:00 pm and 5:00 pm. For each session, one out of four bulls per pen was measured twice, yielding 40 individual CH₄ profiles. Each measurement lasted 3 min, with CH₄ concentrations recorded every 0.5 s (~360 data points per measurement). In cases where an animal did not remain in its position for the full 3 min, the measurement was repeated until a complete 3-min recording was available. The measurement unit (ppm × m) represents the CH₄ concentration (in parts per million) within the measurement distance in meters from the tip of the LMD device to the target and quantifies the methane content along this path. Bulls were typically attracted by licking bowls, which minimized their movement during measurement. The LMD was positioned approximately 1 m from the bull’s snout, with the distance confirmed using an infrared laser distance meter (Bosch, Stuttgart, Germany). CH₄ concentrations were condensed by calculating the arithmetic mean of the local expiration peak values, following the method outlined by Sorg et al. (2018). The LMD has a detectable range of 1 to 50,000 ppm × m (up to 5 vol-%), with an accuracy of approximately ± 10 ppm within a temperature range of -17 to + 50 °C. The device’s auto-calibration feature uses an internal reference cell. External environmental factors such as temperature, humidity, and wind speed were recorded during each measurement.

### Statistical Analyses

All data were analyzed using a repeated-measures model in SAS 9.4 (SAS Institute Inc., Cary, NC). Fixed effects included dietary treatment (CONVL vs. ByProd), time, and their interaction. Random effects consisted of the pen as the experimental unit for variables such as DMI, ME intake, nutrient intake, and individual animals for variables such as ADG and BW. Initial BW was used as a covariate for the final weight. The model followed the methods outlined by St-Pierre (2007), Tempelman (2009), and Bell et al. (2016). A compound symmetric covariance structure was selected based on the Bayesian information criterion, as it provided the best fit among tested structures (compound symmetric, autoregressive type 1, and unstructured). Residuals were tested for normality using the Shapiro-Wilk test, and variables deviating from normality were log-transformed (base 10). Once log-transformed, variables met the assumptions of normality. Statistical significance was set at *P* ≤ 0.05, with trends identified for *P* values between 0.05 and 0.10.

### CH₄ Emission Metrics

Differences in peak CH₄ levels between the dietary treatments were evaluated using the Wilcoxon signed-rank test. CH₄ levels were analyzed in three phases: pre-supplementation (P1: Days 29–135), during supplementation (P2: Days 155–241), and post-supplementation (P3: Days 263–306). Peak CH₄ values (ppm × m), representing eructations and respiration, were integrated using the method described by Sorg et al. (2018), where the arithmetic mean of CH₄ concentration peaks, referred to as peak means (p_means), was calculated. Daily CH₄ emissions (g/d) per animal were estimated by converting the CH₄ peak means (pmean, in ppm × m) using the linear conversion method described by Sorg et al. (2018):

A. $CH_{4}\left(g/d\right)=\;0.78\;\times \;p_{mean}+36.1$

In addition to these assessments, the following formulas have been suggested for estimating daily CH₄ emissions from cattle during the rearing and fattening phases:

B. $CH_{4}\;\left(g/d\right)=\frac{0.065\times GE\;\left(MJ\right)}{0.05565}$ (IPCC, 2006)
C. $CH_{4}\left(g/d\right)=\frac{0.045\;\times \;GE\;\left(MJ\right)}{0.05565}$ (Van Lingen et al. 2019)
D. $CH_{4}\left(g/d\right)=\text{-}102+DMI\;\left(kg\right)\;$× 11.6 + aNDFom (%)×3.74 (Van Lingen et al. 2019)
E. $\begin{array}{lllll} & CH_{4}\;\left(g/d\right)=\;\text{-}102\;+\;DMI\;\left(kg\right)\;\; & \times \;11.6\;+\;aNDFom\;\left(\;\%\;\right)\;\; & \times \;3.74\;--\;EE\;\left(\;\%\;\right)\;\times \;11.1\;+\;\; & BW\left(kg\right)\;\times \;0.164\; \end{array}$ (Van Lingen et al. 2019)

where

GE: Gross energy consumption in MJ per animal per day;

DMI: Dry matter intake in kg per day;

aNDFom: Neutral detergent fiber, amylase-treated and ash-free, expressed as a % of dry matter;

EE: Ether extract, expressed as a % of dry matter;

BW: Body weight of the animal in kg.

These equations were used to contrast the real-time CH₄ measurements obtained with the LMD device.

### Economic Evaluations

To obtain diet/performance-specific income over feed cost (IOFC) results, the required animal biology, ecology, and management data for the CNCPS diet evaluation (Fox et al., 2004) were obtained from the Educational and Research Centre for Animal Husbandry Hofgut Neumühle (Münchweiler, Germany). After 306 d of fattening, bulls were slaughtered. The diet/performance-specific IOFC for the 306-day fattening period was calculated as follows:


$$\begin{array}{l} IOFC=\;\gamma \;\times \;d_{(\;\%\;)}\;\times \;\mu \;\text{-}\;\delta \; \end{array}$$

where:

γ: average live weight in kg;

_d(%):_ dressing percentage;

µ: meat price in €, adjusted for the classification/conformation premium;

δ: dietary costs in €.

To accommodate a flexible and individual price scheme for carcasses, the meat prices were adjusted within the framework of a bonus/malus according to the EU classification of carcass quality. Specifically, the meat/carcass price (€) was determined by the base price (classification of the animal type, e.g., calf or young bull) plus or minus the conformation premiums (EUROP classification) and the fat class premiums (1 to 5 classification). The prices for agricultural products were taken from the Board of Trade data for 2022 (Statista 2024; https://de.statista.com). Standard market data from February 2023 reported by VEZG (2023; https://www.vezg.de/vereinigungspreis-jungbullen-r3-und-o3-1.html) was used for the calculations of carcass base prices and premiums. In addition, the calculations of excreta and nutrient availability were performed according to the Cornell Net Carbohydrate and Protein System (CNCPS) as described by Fox et al. 2004.

## RESULTS

### Intake and Growth Performance

**Figure 1.**
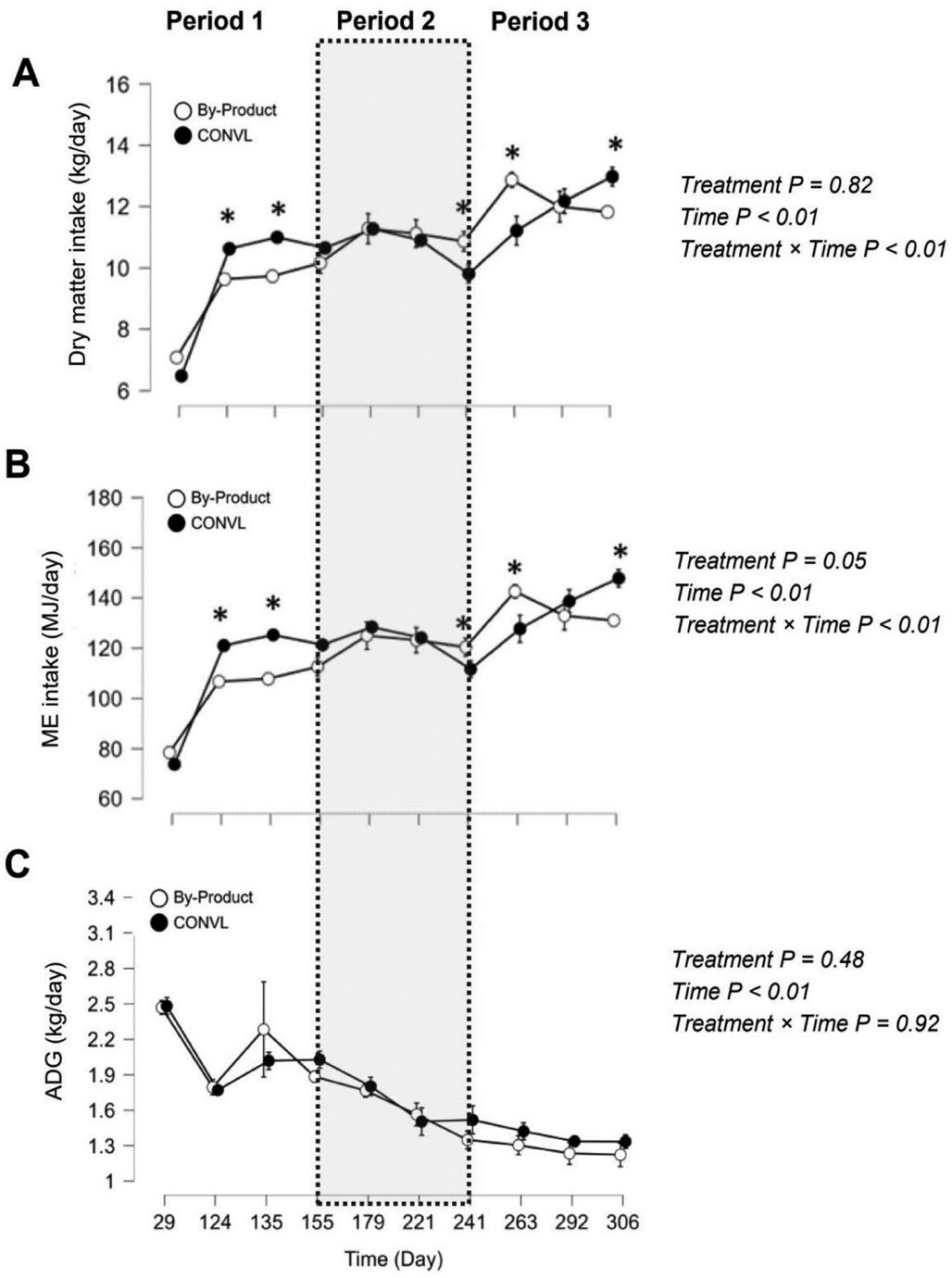
(A) Dry matter intake, (B) metabolizable energy (ME) intake, and (C) average daily gain (ADG) of Simmental bulls fed two types of total mixed rations (TMR): one based on byproducts and co-products (ByProd) and a conventional ration based on silage (CONVL). Asteriscs indicate a difference (*P < 0.05) between the groups at a given time (day). Data are presented as means ± SEM. The shaded areas represent the data divided into the periods before the ClimaSAN supplementation (Period 1; days 29-135), during the ClimaSAN supplementation (Period 2, days 155-241) and after the ClimaSAN supplementation (period 3, days 263-306).

**Figure 2.**
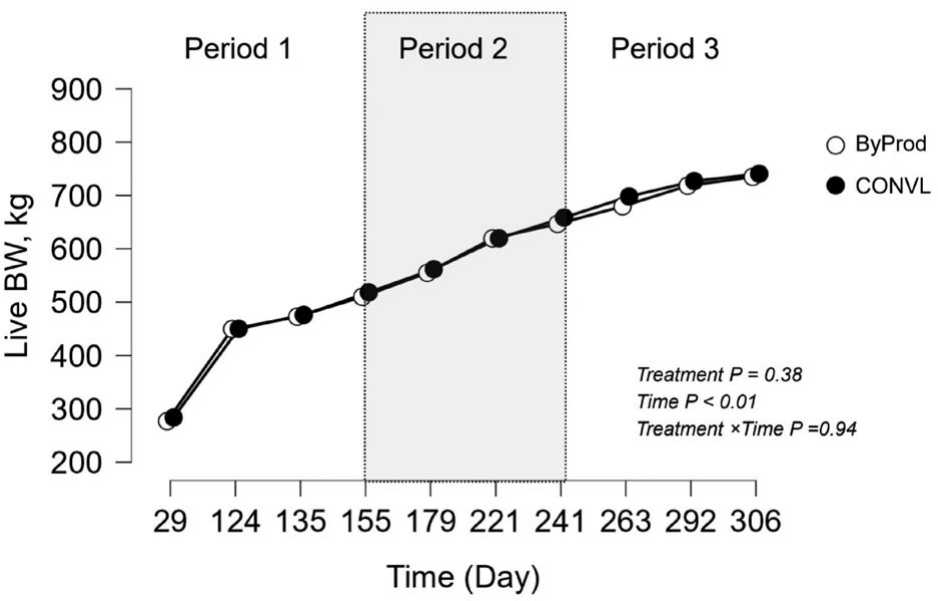
Live body weight of Simmental bulls fed two types of total mixed rations (TMR): one based on byproducts and co-products (ByProd) and a conventional ration based on silage (CONVL). Data are presented as means ± SEM. The shaded areas represent the data divided into the periods before the ClimaSAN supplementation (Period 1; days 29-135), during the ClimaSAN supplementation (Period 2, days 155-241) and after the ClimaSAN supplementation (period 3, days 263-306).

The results of nutrient intake and growth performance are shown in Table 3 and Figures 1 and 2. Intakes of DM (Figure 1A), starch, and uNDF were affected by time and the interaction between time and treatment (*P* < 0.01). On days 124, 135, and 306, DMI was significantly higher in the CONVL group, while on days 241 and 262, it was significantly lower compared to the ByProd group. Intakes of ME, aNDFom, ADF, ADL, peNDF, crude fiber, NFC, sugar (WSC), soluble fiber, CP, soluble protein, and EE were affected by dietary treatments (CONVL vs. ByProd), time, and the interaction among them (*P* < 0.01). On days 124, 135, and 306, ME intake (Figure 1B) was significantly higher in the CONVL group, while on days 241 and 262, it was significantly lower compared to the ByProd group. Bulls fed the ByProd-TMR in our study consumed significantly less ME (-3.9 MJ/d), aNDFom (-204 g/d), ADF (-290 g/d), ADL (-13 g/d), peNDF (-577 g/d), crude fiber (-563 g/d), CP (-99 g/d), soluble protein (-94 g/d), and EE (-128 g/d) than bulls fed the CONVL TMR. Conversely, intakes of NFC (+233 g/d), sugar (WSC) (+422 g/d), and soluble fiber (+41 g/d) were significantly higher in the ByProd-TMR group compared to the CONVL-TMR group. Although the two dietary treatments differed in moisture content by 7% units (Table 1) and in particle size distribution (Table 2), the sorting index values were not significantly different between the treatments (*P* > 0.10).

**Table 3.** Feed intake and growth performance of fattening bulls fed a ration composed of universally available by-/and co-products (ByProd) or a conventional silage-based ration (CONVL) total mixed rations (TMR) during 306 days on trial

Item^1^	Treatments		SEM	*P*-value		
	ByProd	CONVL		Treatment	Time	Treatment × Time
Feed DMI, kg/d	10.7	10.7	0.09	0.82	<0.01	<0.01
ME intake, MJ/d	118	122	1.00	0.05	<0.01	<0.01
Crude fiber intake, kg/d	1.08	1.64	0.19	<0.01	<0.01	<0.01
aNDFom intake, kg/d	3.36	3.56	0.29	<0.01	<0.01	<0.01
ADF intake, kg/d	1.75	2.04	0.18	<0.01	<0.01	<0.01
ADL intake, g/d	298	311	2.60	0.02	<0.01	<0.01
peNDF intake, kg/d	1.77	2.35	0.23	<0.01	<0.01	<0.01
NFC intake, kg/d	4.84	4.62	0.40	0.01	<0.01	<0.01
Sugar (WSC) intake, g/d	878	456	1.22	<0.01	<0.01	<0.01
Starch intake, kg/d	3.26	3.26	0.27	0.82	<0.01	<0.01
Soluble fiber intake, g/d	497	457	4.10	<0.01	<0.01	<0.01
uNDF intake, g/d	813	811	6.70	0.72	<0.01	<0.01
Crude protein (CP) intake, kg/d	1.37	1.47	0.12	<0.01	<0.01	<0.01
Soluble Protein intake, g/d	438	532	4.80	<0.01	<0.01	<0.01
Ether Extract (EE) intake, g/d	303	431	4.60	<0.01	<0.01	<0.01
Live BW, kg	564	572	8.26	0.38	<0.01	0.94
ADG, kg/d	1.70	1.72	0.04	0.48	<0.01	0.92
ADG/DMI ratio	0.15	0.15	0.004	0.57	<0.01	0.23
ADG/MEI ratio	0.02	0.02	0.001	0.58	<0.01	0.96
CH₄ peaks (ppm × m) ^2^						
P1	107	108	4.17	0.69	0.01	0.28
P2	100	95	3.03	0.43	0.02	0.52
P3	107	110	3.98	0.76	<0.01	0.10
Overall	104	103	2.14	0.62	<0.01	0.21

^1^Abbreviations: Feed DMI (feed dry matter intake), ME intake (metabolizable energy intake), aNDFom (neutral detergent fiber, organic matter basis), ADF (acid detergent fiber), ADL intake (acid detergent lignin intake), peNDF (physically effective neutral detergent fiber), NFC (Non-fiber carbohydrate), sugar (WSC) (water-soluble carbohydrate), uNDF (undigested neutral detergent fiber), CP (crude protein), EE (Ether extract), Live BW (live body weight), ADG (average daily gain), ADG/DMI ratio (ratio of average daily gain to dry matter intake), ADG/MEI ratio (ratio of average daily gain to metabolizable energy intake), P1 (pre-supplementation period), P2 (supplementation period), P3 (post-supplementation period), and overall (entire experimental period).

^2^Table presents average CH_4_ peak mean results (ppm × m) as dietary bull-group individual CH_4_ emission point estimator, categorized by three phases: Pre-supplementation (Period 1; P1, day 29-135), During supplementation (Period 2; P2, Day 155-241), and Post-supplementation (Period 3; P3, day 263-306), along with the overall average from day 29-306.

Growth metrics such as ADG (Figure 1C), ADG/DMI ratio and ADG/MEI ratio showed no significant differences between treatments, indicating consistent growth rates across dietary treatments despite differences in DMI and ME intake. Initially, ADG remained well above 1.7 kg/d in both groups until day 179, after which it settled to 1.3 kg/d between day 179 and day 306. Live BW (Figure 2) was affected by time (*P* < 0.01) but remained unaffected by the dietary treatments (CONVL vs. ByProd) and their interaction. Both groups showed numerically similar total live weight gains (529 kg/head over 306 d).

### Fattening bull enteric CH₄ emissions

**Figure 3.**
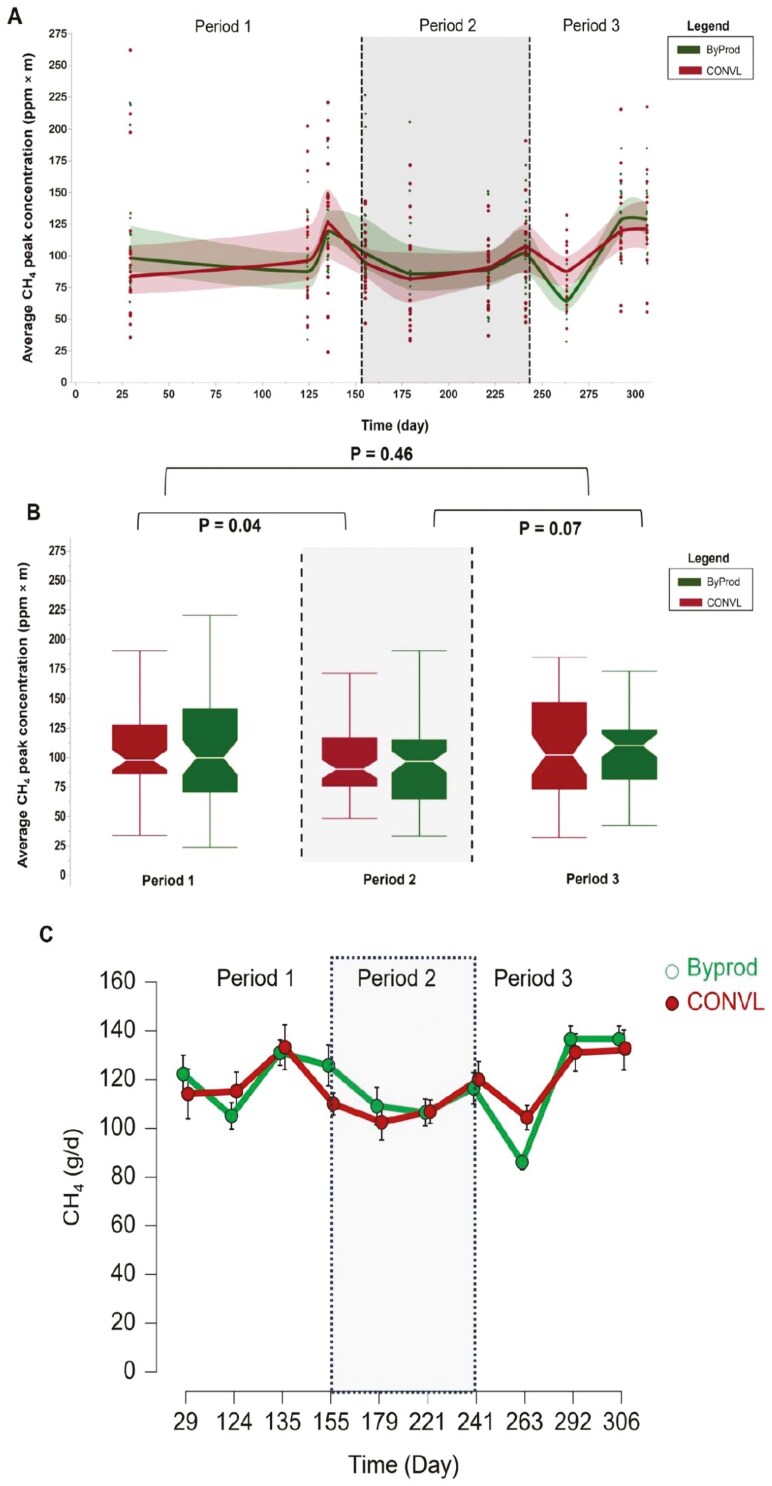
Panel A depicts the average methane (CH_4_) peak mean (p_mean) concentration for Simmental bulls over a 306-day trial. The bulls were fed two types of total mixed rations (TMR): one based on by- and co-products (ByProd) and the other a conventional silage-based ration (CONVL). The scattered data points represent daily methane emission peaks. Panel B displays box plots that detail median values and interquartile ranges of CH₄ emissions, illustrating the impact of dietary regimes across three distinct phases. Overlaid *P*-values provide a statistical evaluation of the differences between each consecutive period (Period 1 vs Period 2, Period 2 vs Period 3, and Period 1 vs Period 3). Panel C depicts the average methane (CH_4_ g/day) concentration for Simmental bulls over a 306-day trial. The shaded areas represent the data divided into the periods before the ClimaSAN supplementation (Period 1; days 29-135), during the ClimaSAN supplementation (Period 2, days 155-241) and after the ClimaSAN supplementation (period 3, days 263-306).

Figure 3A shows significant temporal variations in peak concentrations of CH₄ (ppm × m). These fluctuations were not significantly affected by dietary schedule (CONVL vs. ByProd) or by a time-treatment interaction (Table 3). The boxplot in Figure 3B shows the CH₄ peaks across 3 different phases of the study. It shows a significant decrease of approximately 9.82% (*P* = 0.04) during the ClimaSAN complex supplementation phase (P2) compared to the pre-supplementation phase (P1), a pattern that is consistent across diets. After the discontinuation of ClimaSAN (P3), there was a trend indicating an increase in CH₄ emissions of approximately 12.5% compared to the supplementation phase (P2), although this increase did not finally reach statistical significance (*P* = 0.07). Importantly, even with the small rise in CH₄ emissions of 1.42% in P3, the values did not exceed those observed in the initial phase (P1; *P* = 0.46). Figure 3C shows significant temporal variations in concentrations of CH₄ (g/d).

**Figure 4.**
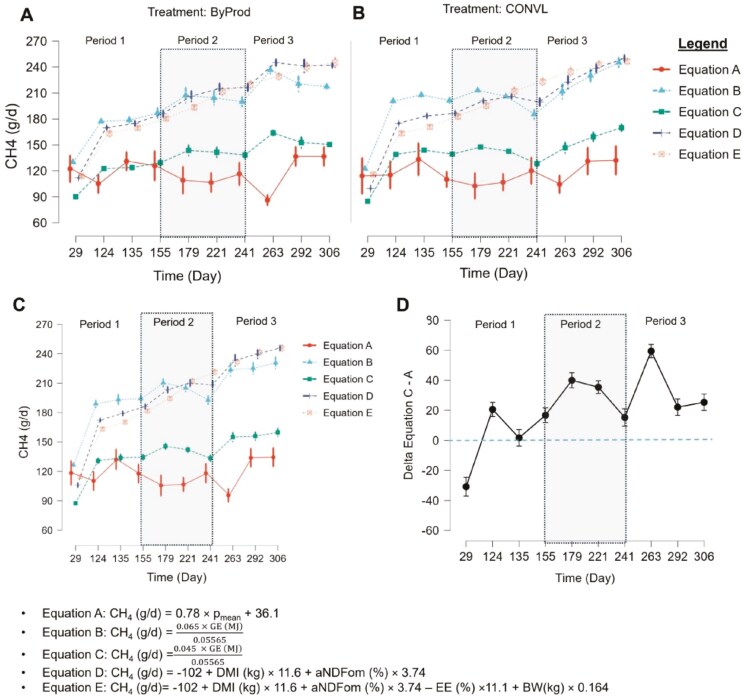
Comparative analysis of methane (CH₄) emissions from Simmental bulls fed two types of total mixed rations (TMR): one based on byproducts and co-products (ByProd) and a conventional ration based on silage (CONVL). The shaded areas represent the data divided into the periods before the ClimaSAN supplementation (Period 1; days 29-135), during the ClimaSAN supplementation (Period 2, days 155-241) and after the ClimaSAN supplementation (period 3, days 263-306). Panel A and Panel B show emissions for the ByProd and CONVL treatments, respectively, over three experimental phases using different equations (A-E), distinguished by color and style. Panel C summarizes the emission data for both treatments, allowing a comparative analysis. Panel D shows deviations (delta values) between equations C and A. Emissions are expressed in g per day, derived from direct real-time measurements with a portable laser methane detector.

Figure 4 shows a comparative analysis of CH₄ emissions (g/d) from bulls across the three experimental phases using five different prediction equations (A to E). Emission data for the ByProd and CONVL feeds are shown in Figures 4A and 4B, respectively. An aggregated view of the average CH₄ emissions from both treatments is shown in Figure 4C. It is noteworthy that equations B, D and E tended to overestimate CH₄ emissions compared to the direct measurements (equation A). Conversely, equation C closely matched the direct measurements, which was particularly noticeable in the pre-supplementation phase. Figure 4D highlights the discrepancies (delta values) between equations C and A, which show significant deviations during the supplementation and post-supplementation phases.

### Carcass traits and dressing percentage

When carcass data for the ByProd and CONVL TMR groups were examined, no significant effects on carcass revenue per kg or percent dressing were observed (Table 4). Carcass revenue for both treatment groups ranged from €4.55/ kg to €4.80/ kg, and dressing percentage ranged from 56.0% to 58.0% (Table 4).

**Table 4.** Economic performance of fattening bulls fed a ration composed of universally available by-/and co-products (ByProd) or a conventional silage-based ration (CONVL) total mixed rations (TMR) after 306 days on trial

Item	Treatments		SEM	*P*-value
	ByProd	CONVL		
Final live weight (kg/head at d 306)	735	741	10.9	0.60
Total live weight gain (kg/head during 306 d)	529	529	10.8	0.81
Total Dry matter feed intake (kg/head)	2961	2925	34.1	0.33
Total as-fed feed intake (kg/head)	5684	6489	58.3	<0.001
Dressing (%)	57.0	56.8	0.01	0.71
Carcass income (€/kg)	4.61	4.60	0.05	0.80
Income (€/head)	1931	1936	25.0	0.80
Feed cost (€/head)	710	749	8.3	0.005
Income over feed costs (€/head)	1221	1187	23.4	0.11
Profit margin (€/head)	371	337	23.4	0.11

### Economic analysis of fattening bulls on ByProd and CONVL diets

The economic performance of fattening bulls fed either ByProd or CONVL was evaluated over 306 d. As shown in Table 4, there were no significant differences in final live weight, total weight gain or overall carcass income between the two dietary treatments, indicating comparable growth performance. The initial investment costs for each young bull averaged €850, with an average initial weight of 209 kg. Total direct costs, including feed, were slightly higher in the CONVL group (€1,599 per animal) than in the ByProd group (€1,560 per animal) due to the slightly higher feed costs. While feed efficiency and ADG were the same between the groups, the ByProd diet had a cost advantage. Specifically, feed costs were lower in the ByProd group (€710 per animal) than in the CONVL group (€749 per animal), resulting in a statistically significant difference (*P* = 0.005). Nevertheless, income over feed costs (IOFC) and profit margins were similar between groups, indicating that both feeding strategies are economically viable. Overall, while ByProd feeding appears to offer a slight economic advantage due to lower feed costs, both feeding strategies provide substantial returns with an equal return on investment (ROI) of 1.25, making both options suitable depending on available resources and specific farm management objectives.

## Discussion

Efficient cattle fattening plays a crucial role in livestock management, aiming to maximize weight gain while balancing economic and environmental concerns. This study evaluated the impact of two Total Mixed Ration (TMR) diets (CONVL and ByProd) on various parameters, including nutrient intake, growth performance, CH₄ emissions, carcass characteristics, and economic outcomes in Simmental fattening bulls. The results revealed significant temporal variations in dry matter, starch, and uNDF intake, which likely reflect complex physiological responses influenced by growth dynamics and hormonal changes as the bulls mature. Bulls fed the ByProd-TMR consumed less metabolizable energy (ME), fiber components (aNDFom, ADF, ADL, peNDF, and crude fiber), crude protein (CP), soluble protein, and ether extract (EE), while their intake of non-fiber carbohydrates (NFC), water-soluble carbohydrates (WSC), and soluble fiber was significantly higher. These differences suggest that the ByProd-TMR, containing ingredients such as beet pulp silage, brewers’ grains, wheat bran, wheat gluten feed, and beet molasses mix, may be lower in energy and fiber but richer in easily fermentable carbohydrates. Despite these nutritional differences, key growth performance indicators like live weight gain and average daily gain (ADG) were unaffected. Initial ADG exceeded 1.6 kg/d and stabilized between 1.3 and 1.6 kg/d between days 179 and 306, with both groups achieving similar total live weight gains. This aligns with previous research showing that different feed formulations, even with varied nutritional profiles, can produce comparable growth outcomes. Studies have highlighted ADG as a critical factor in the economic efficiency of fattening bulls (Yan et al., 2009; Garip et al., 2010; Schönleben et al., 2020; Liu et al., 2021), further supported by data from Simmental bulls on high-energy diets reporting daily gains of 1.21 kg/d (Schwarz et al., 1992) and peak gains of 1.54 g/d (Schwarz and Kirchgessner, 1990). Our recent study with Simmental bulls similarly showed ADG of 1.87-1.84 kg over 272 d with dry or corn silage-based TMR diets (Koch et al., 2023). This suggests that alternative feed formulations, such as byproducts, can be used without compromising growth performance.

CH₄ emissions from livestock are a key focus in climate change mitigation (Hristov et al., 2022). In cattle, CH₄ is produced during the digestion of cellulose and hemicellulose through enteric fermentation, driven primarily by methanogenic Archaea (McAllister and Newbold, 2008). While respiration chambers are considered the gold standard for CH₄ measurement (Donoghue et al., 2016a, 2016b; Sorg et al., 2018), their use is limited by practical constraints. More feasible, large-scale methods, such as the GreenFeed system or laser CH₄ detectors (LMD), have emerged as effective alternatives for on-farm measurements. The LMD, in particular, has demonstrated strong correlations with the GreenFeed system (*rp* = 0.66), with further improvement when used alongside predictive models (*rp* = 0.74) (Sorg et al., 2018), making it a valuable tool for large-scale, real-world applications. In this study, we observed significant temporal variations in CH₄ emissions, with the ClimaSAN supplement—a blend of tannin and seaweed extracts—resulting in a substantial reduction in emissions, regardless of diet. These findings align with previous research on the CH₄-reducing potential of tannin and seaweed extracts in ruminants (Goel and Makkar, 2012; Machado et al., 2015; Cardoso-Gutierrez et al., 2021). The notably lower post-ClimaSAN CH₄ emissions in Period 3 compared to the baseline (Period 1) suggest the potential for long-term CH₄ reduction, likely due to sustained adaptations in the rumen microbiome. However, the observed increase in CH₄ emissions after the ClimaSAN administration highlights the need for frequent supplementation to maintain reduced emission levels. It is important to note that due to the study design, a true control group (i.e., animals that did not receive ClimaSAN) was not included during the supplementation period. As a result, potential undetected confounding factors may have influenced the observed reduction in CH₄ emissions. Although the reduction of approximately 10 % is promising, these results should therefore be interpreted with caution.

Carcass characteristics were notably similar between the ByProd and CONVL groups, suggesting that alternative but balanced feeding strategies can yield comparable carcass qualities. Feed efficiency was also comparable, indicating that beef producers can select from a range of feeding options without compromising animal performance, provided the diets are correctly balanced. This flexibility is economically advantageous, as feed costs account for a significant portion of total beef production expenses. The comparable return on investment (ROI) between both groups further supports the economic viability of these feeding strategies, with the ByProd group showing potential for higher net profitability due to initial feed cost savings.

A comparative analysis of CH₄ emissions using different prediction equations (B-E) revealed discrepancies between theoretical estimates and direct measurements. Direct measurements (equation A) consistently yielded lower CH₄ values than the theoretical equations, with equation C showing the closest agreement, particularly during the pre-supplementation period (Period 1). However, significant deviations between equations C and A during and after ClimaSAN supplementation (Periods 2 and 3) highlight the complexities of accurately predicting CH₄ emissions. These findings emphasize the importance of selecting appropriate prediction methods and validating them under varying dietary and management conditions to ensure their accuracy. This is crucial for developing reliable greenhouse gas inventories and assessing mitigation strategies in livestock systems.

## Conclusions

In light of the pressing need to reduce greenhouse gas emissions from ruminant livestock systems, our study suggests that bull fattening can play a meaningful role in mitigation efforts. Our findings align with prior research on the use of tannin- and seaweed-based supplements, confirming their potential to reduce enteric CH₄ emissions. The addition of ClimaSan reduced CH₄ emissions by 9.82%, irrespective of the diet, and indicated a long-term CH₄ mitigation effect, likely due to the sustained adaptation of the rumen microbiome. These results emphasize the importance of validating CH₄ prediction models across various dietary conditions with direct measurements to ensure their reliability and applicability in real-world scenarios. Amid climate change and the growing need to prioritize food crop production, dietary strategies incorporating byproducts offer a viable solution. The byproduct-dominated diet in this study resulted in growth performances that were biologically and economically comparable to the conventional diet, offering flexibility in feed sourcing without compromising productivity. This flexibility has significant economic benefits, as demonstrated by the similar return on investment for both dietary groups. In conclusion, feeding strategies based on byproducts and co-products from the human food industry present promising alternatives for fattening bulls. Given that trials with fattening bulls are less complex than those with dairy cows, these findings may serve as a model for dairy systems. The significant CH₄ emission reductions observed with the ClimaSan supplement further underscore its potential for broader application in reducing emissions across various ruminant systems.
